# Rad5 Template Switch Pathway of DNA Damage Tolerance Determines Synergism between Cisplatin and NSC109268 in *Saccharomyces cerevisiae*

**DOI:** 10.1371/journal.pone.0077666

**Published:** 2013-10-10

**Authors:** Dilip Jain, Wolfram Siede

**Affiliations:** Department of Cell Biology and Anatomy, University of North Texas Health Science Center, Fort Worth, Texas, United States of America; University of Minnesota, United States of America

## Abstract

The success of cisplatin (CP) based therapy is often hindered by acquisition of CP resistance. We isolated NSC109268 as a compound altering cellular sensitivity to DNA damaging agents. Previous investigation revealed an enhancement of CP sensitivity by NSC109268 in wild-type *Saccharomyces cerevisiae* and CP-sensitive and -resistant cancer cell lines that correlated with a slower S phase traversal. Here, we extended these studies to determine the target pathway(s) of NSC109268 in mediating CP sensitization, using yeast as a model. We reasoned that mutants defective in the relevant target of NSC109268 should be hypersensitive to CP and the sensitization effect by NSC109268 should be absent or strongly reduced. A survey of various yeast deletion mutants converged on the Rad5 pathway of DNA damage tolerance by template switching as the likely target pathway of NSC109268 in mediating cellular sensitization to CP. Additionally, cell cycle delays following CP treatment were not synergistically influenced by NSC109268 in the CP hypersensitive *rad5Δ* mutant. The involvement of the known inhibitory activities of NSC109268 on 20S proteasome and phosphatases 2Cα and 2A was tested. In the CP hypersensitive *ptc2Δptc3Δpph3Δ* yeast strain, deficient for 2C and 2A-type phosphatases, cellular sensitization to CP by NSC109268 was greatly reduced. It is therefore suggested that NSC109268 affects CP sensitivity by inhibiting the activity of unknown protein(s) whose dephosphorylation is required for the template switch pathway.

## Introduction

Since its discovery three decades ago, Cisplatin (CP) has been widely used as an effective anticancer agent against a wide variety of solid tumors like tumors of ovary, testis, head and neck, cervix and lung [1,2]. However, treatment success by platinum agents is diminished by both intrinsic and acquired resistance, necessitating a dose escalation that is limited by side effects like nephrotoxicity, ototoxicity, peripheral neuropathy and myelosuppression [1,3,4]. Acquired resistance is often multifactorial in nature, with common mechanisms attributed to decreased cellular drug accumulation through reduced influx or increased efflux [5,6], elevated thiol content and increased ability to repair or tolerate platinum DNA adducts [3,4]. A non-toxic compound used in combination with CP that potentiates sensitivity may increase the therapeutic index of CP, especially in the case of otherwise CP resistant cancers [7-10]. 

A two-hybrid yeast assay was used by us to screen the National Cancer Institute’s Diversity Set for compounds that can modify the checkpoint response elicited by the topoisomerase I inhibitor camptothecin [11]. Initially, NSC109268 was isolated as such an agent that reduces the checkpoint response to camptothecin. On further study by quantitative survival analysis, it was revealed that NSC109268 *increased* cellular CP sensitivity [12]. This was in marked contrast to its general tendency of *decreasing* sensitivity to other DNA damaging agents like nitrogen mustard [12]. CP and nitrogen mustard both create common lesions, namely interstrand crosslinks but with different yield.

NSC109268 had been shown to inhibit the chymotrypsin-like activity of the 20S proteasome in both Jurkat T cell extract and rabbit purified 20S proteasomes using an *in vitro* assay [13]. In addition, inhibition of phosphatases by NSC109268 had been suspected following molecular modeling, using the human PP2Cα structure in a virtual ligand screening of the Diversity Set of compounds [14]. Using biochemical assays of enzyme activity, NSC109268 was indeed found to strongly inhibit PP2Cα and, less severely, the PP2A group of serine-threonine protein phosphatases [14]. 

Described initially in budding yeast, we further confirmed cellular sensitization to CP by NSC109268 in the CP-sensitive ovarian carcinoma cell line 2008 and, even more pronounced, in its CP-resistant counterpart, 2008/C13 [12]. Cellular sensitization to CP by NSC109268 was consistently correlated with a slower S to G2/M phase progression in both yeast and the CP-resistant carcinoma cell line [12]. Although NSC109268 enhanced CP-induced p53 levels, its effect on cell death following CP (i.e. apoptosis and necrosis) was not dependent on p53 [15].

 Given the similarities of the effect of NSC109268 on mediating cellular sensitization to CP in yeast and cancer cell lines, given also the high degree of conservation of DNA repair pathways and the availability of a collection of deletion mutants of non-essential yeast genes, yeast must be considered a valuable model to study the targeted pathway(s) of NSC109268 that are relevant for CP sensitivity [16]. The major target of CP is chromosomal DNA, with the majority of CP adducts comprising of DNA intrastrand crosslinks, mainly diguaninyl crosslinks [17]. Albeit much less frequently, CP also induces the relatively much more lethal interstrand crosslinks [18]. Nucleotide Excision Repair (NER) is the major pathway for bulky platinum adduct removal and thus error-free repair of DNA damage by CP [4]. Consequently, defects in the NER pathway result in hypersensitivity to platinum agents and restoration of NER integrity correlates with reversal of CP sensitivity [19]. Increased expression of the NER gene *ERCC1* (*RAD10* in budding yeast) is frequently associated with CP resistance in ovarian and gastric tumors [1].

Interestingly, among various predictors of CP sensitivity examined, such as increased platinum accumulation, decreased glutathione levels, decreased adduct removal or decreased tolerance to platinum-DNA adducts, decreased tolerance was the strongest predictor of CP sensitivity in ovarian cancer cell lines [20]. Furthermore, 2008/C13 cells have been described as being more efficient in replicative bypass of CP lesions than their CP-sensitive counterparts [21]. Therefore, inhibitors of specific DNA repair or tolerance pathways might prove especially efficacious when used in combination with CP. 

Activities of polymerase ζ, polymerase η and RAD18, all involved in control of various modes of DNA damage tolerance are required for replicative bypass of CP intrastrand crosslinks [22]. In response to DNA damage, yeast Proliferating Cell Nuclear Antigen (PCNA) is monoubiquitinated or polyubiquitinated at K164. Monoubiquitination of PCNA by RAD18, a ubiquitin ligase, promotes translesion synthesis mediated by Polζ or Polη [23,24]. During translesion synthesis, low fidelity DNA polymerases replicate directly past the lesion in either an error-prone or error-free fashion. Human cells expressing no Polη or reduced levels of REV3, an essential component of the translesion polymerase DNA Polζ, are more sensitive to CP [25-27]. 

K63-linked polyubiquitination, extending monoubiquitinated K164 of PCNA, depends on Ubc13–Mms2 (forming the E2 enzyme) and Rad5 (the E3 enzyme) and is required for an error-free damage tolerance pathway, most likely mediated by template switch (TS) (Figure S1) [24,28-31]. In contrast to translesion synthesis, the lesion is avoided by a “copy choice” mechanism using an alternate, undamaged DNA template. Upon DNA damage, a lesion in the leading strand template can lead to uncoupling of leading and lagging strand synthesis and single-stranded DNA gaps can be found in both leading and lagging strands. Subsequently, strand invasion mediated by certain Homologous Recombination (HR) factors that include Rad51, Rad52, the complex of Rad55*-*Rad57, promote the formation of TS intermediates that are dissolved by the action of the Sgs1-Top3 complex (Figure S1) [28,30,31]. 

In this study, using survival and cell cycle analyses, we identified the Rad5 pathway of damage tolerance by TS as the target pathway of NSC109268 relevant for CP sensitization. Inhibition of this pathway appears to result in the observed delayed S phase traversal of CP + NSC109268 treated yeast cells. Furthermore, our data suggest a critical role of NSC109268 as a PP2Cα and PP2A phosphatase inhibitor in influencing cellular sensitization to CP.

## Materials and Methods

### Chemicals

Cisplatin (CP) was purchased from Sigma Aldrich. NSC109268 was initially provided as part of the Diversity Set compound library by the Developmental Therapeutics Branch of the National Cancer Institute and later synthesized by Omm Scientific, Dallas, Texas. NSC109268 was dissolved at a stock concentration of 1 mg/ml in dimethylsulfoxide, CP at 10 mg/ml in dimethylformamide.

### Yeast strains

*S. cerevisiae* BY4741 (MATa *his3Δ1 leu2Δ0 met15Δ0 ura3Δ0*) was the parental haploid wild-type strain used throughout the study unless indicated otherwise. Haploid mutants, each deleted for a defined open reading frame of a non-essential gene, were from the systematic gene deletion collection (purchased from Open Biosystems). BY4741 *rad5∆::HIS3* and BY4741 *sml1∆::KanMX4 rad53∆::URA3* were constructed in our laboratory. Strains YJK17 (*MATα hoΔ hmlΔ::ADE1 hmr∆::ADE1 arg5,6∆::HPH::* MATa *-inc ade1-100 leu2,3-112 lys5 trp1::hisG ura3-52 ade3::GAL::HO*), YJK24 (*ptc2Δ::URA3MX ptc3Δ::NatMX*. YJK17 isogenic), YJK26 (*pph3Δ::KanMX*, YJK17 isogenic) and YJK70 (*pph3Δ::KanMX ptc2Δ::URA3MX ptc3Δ::NatMX*, YJK17 isogenic) were kindly provided by Dr. James Haber [32]. Diploid strain D7 (*MAT*a/*α ade2-119/ade2-40 trp5-12/trp5-27 ilv1-92/ilv1-92 CYH/cyh2*) was originally from Dr. Fritz Zimmermann [33]. Note that this strain does not require adenine and forms white colonies due to intragenic complementation.

### Cisplatin sensitivity assays

All strains used in this study were grown to early logarithmic phase in YPD (1% yeast extract/2% peptone/2% dextrose). Subsequently, cells were washed, resuspended in phosphate-buffered saline (PBS) and treated with CP and NSC109268 at 30°C with constant shaking, typically for 2 h unless indicated otherwise. For strains YJK17, 24, 26 and 70 5 mM phosphate buffer/5 mM sodium chloride (pH 7.0) was used instead of PBS. After treatment, cell suspensions were appropriately diluted and plated onto YPD plates and, in case of D7, also synthetic media plates lacking tryptophan where appropriate. Published protocols and recipes were followed [34]. Plates were incubated at 30°C to allow colony formation. Surviving fractions after drug treatment were calculated by dividing the titer of macrocolony-forming cells in the drug-treated sample by the titer of macrocolony-forming cells in the untreated control sample. Standard deviations are indicated if experiments were repeated at least three times. Otherwise, data of representative single experiments are shown, but in all cases data points were reproduced at least once. Dose Enhancement Factors (DEF) were calculated by determining the ratios of CP doses resulting in 50% or 10% survival, without vs. with inclusion of NSC109268. For DEF calculations, survival values were corrected for killing by NSC109268 alone (usually 10-30%). 

### Synchronization and flow cytometric DNA analysis

Wild-type yeast (BY4741) and isogenic mutant strain *rad5∆* were grown in YPD overnight at 30°C to early logarithmic phase. Cells were then synchronized in G1 using the yeast mating pheromone, α factor, at a final concentration of 10 µg/ml as previously described [12]. After resuspending cells in PBS, 80 µM CP was administered along with 5 µg/ml of α factor for 1 h at 30°C with shaking. Next, cells were washed, resuspended with PBS and α factor and treated for 20 min with 0.7 µM NSC109268. Control samples, containing the appropriate solvent instead of the drug, were incubated in parallel. After treatment, cells were washed and resuspended in fresh YPD to allow for synchronous reentry into the cell cycle. At the indicated time points, samples were collected, fixed in ethanol, sonicated and stained with SYBR Green I (gel staining solution, Lumiprobe) for FACS analysis of DNA content as described [35]. Cellular fluorescence was measured in a FC500 Flow Cytometer (Beckman Coulter Corp.). CXP and ModFit software were used to obtain histograms depicting DNA content versus cell number (of a total of 10,000 cells) and to calculate the fractions of G1, S and G2/M cells.

## Results

### Administration of cisplatin prior to NSC109268 leads to enhancement of cisplatin sensitivity

**Figure 1:**
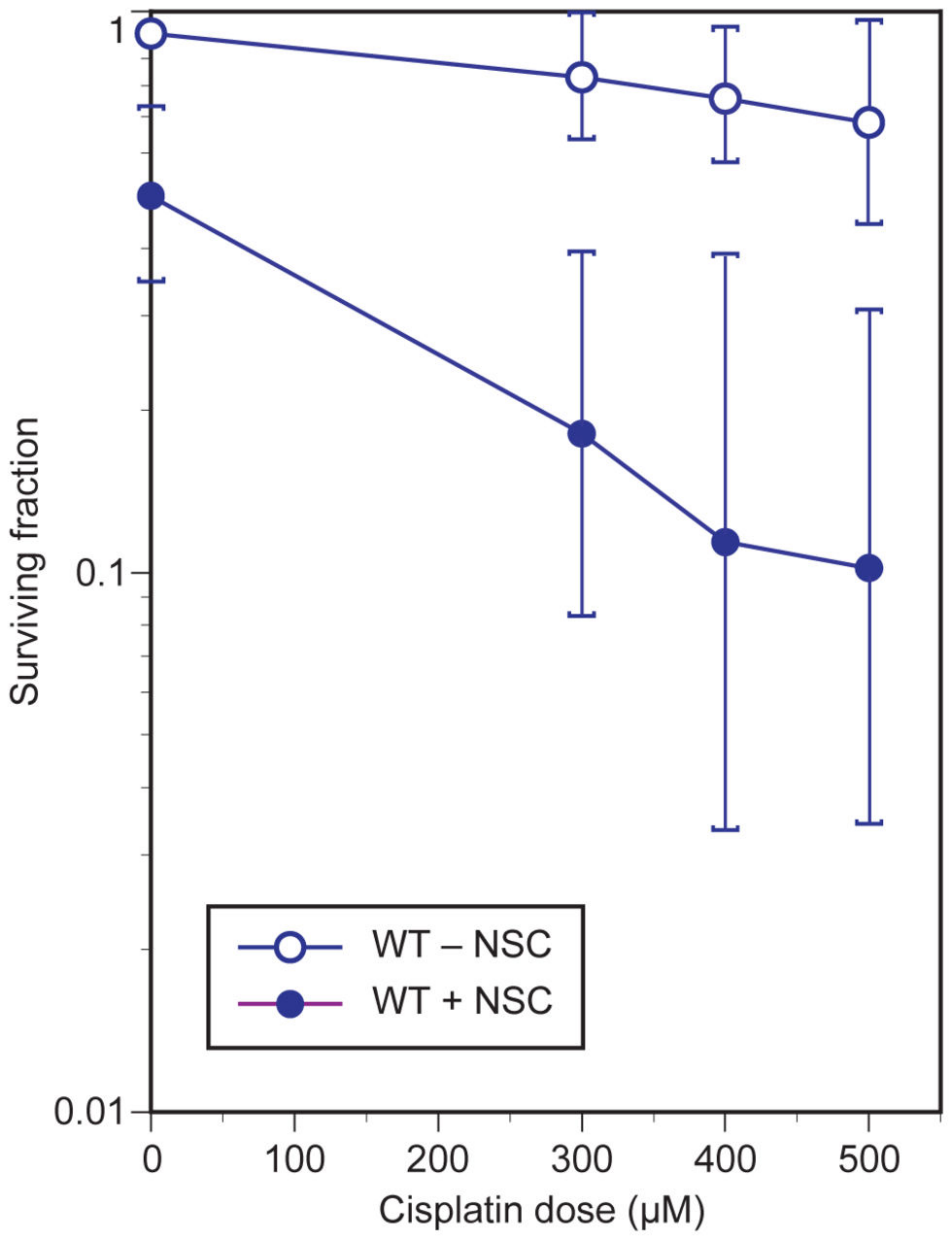
Effect of sequential treatment with cisplatin and NSC109268 on yeast cell killing. Survival of logarithmic-phase haploid wild-type yeast (BY4741) was analyzed after pretreating cells with CP for 1 h, followed by NSC109268 (3 μM) for 1 h in CP-free PBS. Fractions of colony forming cells were plotted as a function of CP dose, with and without NSC109268 administration.

CP uptake is largely mediated by the plasma membrane copper transporter CTR1 in both yeast and mammals [36,37]. Following internalization, both copper and CP were shown to cause downregulation of CTR1 in ovarian cancer cells by the proteasome-mediated pathway [38] contributing to CP resistance. Since NSC109268 may prevent the degradation of CTR1 through its 20S proteasome inhibitory activity [13], we tested CP uptake as a target of NSC109268 in CP sensitization. Haploid wild-type yeast cells were treated with CP for 1 h, then washed to remove CP and treated with NSC109268 for another hour. Following this regimen, NSC109268 clearly sensitized yeast cells to CP (Figure 1) synergistically, as with simultaneous administration of NSC109268 and CP [12] or pretreatment with NSC109268 before adding CP (data not shown). Since NSC109268 sensitized previously CP-treated cells even in the absence of external CP, we conclude that mechanisms other than increased CP uptake, such as inhibition of DNA repair, must be responsible for sensitization to CP. 

### Mutant survey reveals the DNA damage tolerance pathway by template switch as a crucial target of NSC109268 in sensitization to cisplatin

**Figure 2:**
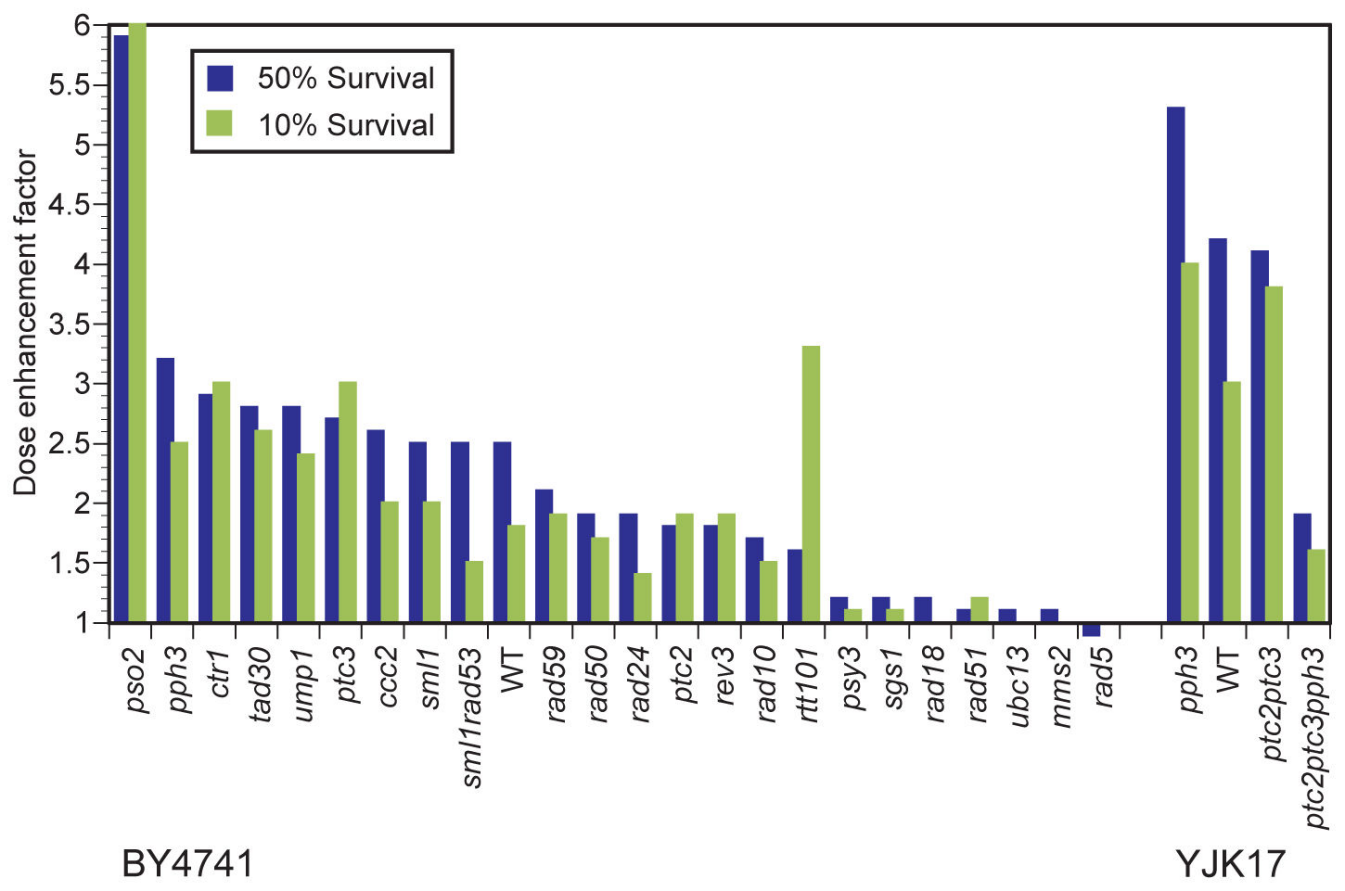
Effect of NSC109268 on cisplatin sensitivity in various deletion mutants. Strains of two different genetic backgrounds (BY4741, YJK17) were ranked by dose enhancement factors at 50% survival. Data were corrected for inactivation by NSC109268 alone. See Table S1 for the putative role of gene products.

**Figure 3:**
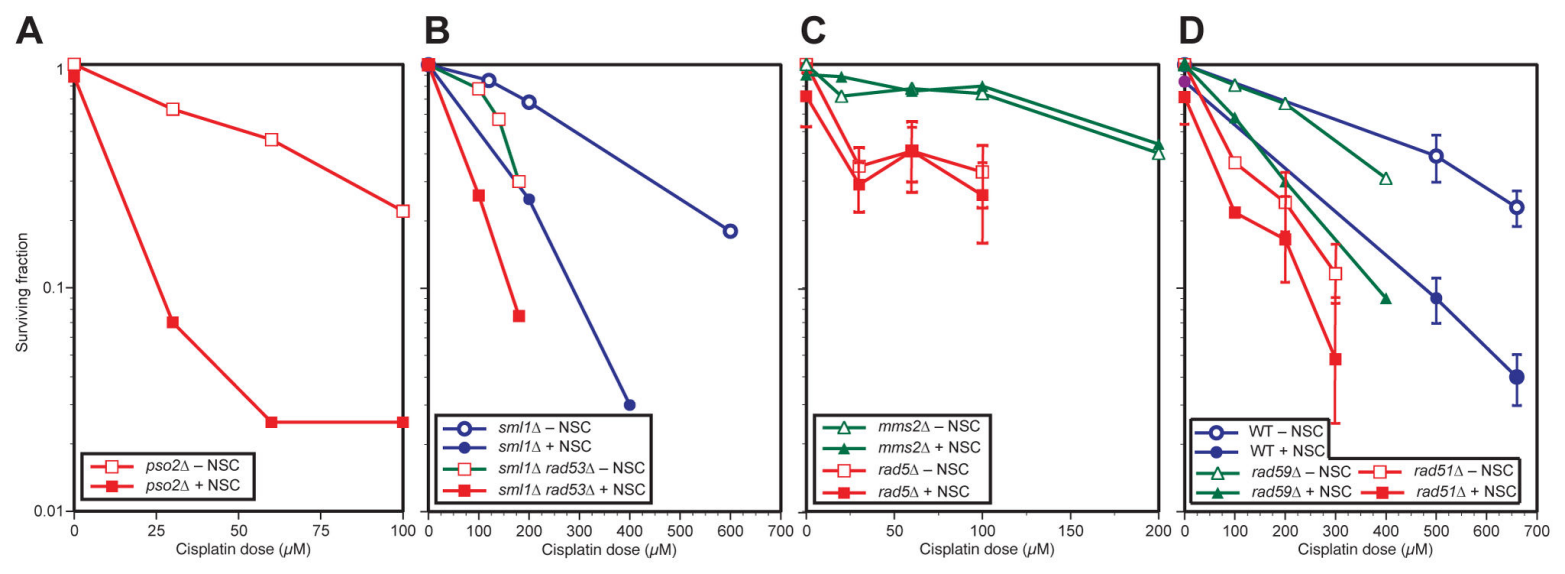
Dose response curves of wild type and various isogenic haploid mutant strains, treated with cisplatin alone or with cisplatin and NSC109268 combined. (A) Survival analysis of *pso∆*, (B) *sml1*∆ and *sml1*∆*rad53*∆, (C) *mms2∆* and *rad5∆*, (D) *rad51*∆ and *rad59*∆ as compared to wild type (BY4741). Surviving fractions of colony forming cells were plotted as a function of CP dose. Logarithmic-phase cells were treated, with or without NSC109268 (3 μM), for 2 h at 30°C. Use of symbols is indicated in the figure. Symbols without error-bars indicate single representative experiments.

In order to identify the pathway targeted by NSC109268 in mediating cellular sensitization to CP we reasoned that, if the critical pathway is already inactive, such mutant cells will be CP sensitive and no sensitization that exceeds an additive effect will be achieved by combination treatment. Several functional screens in *S. cerevisiae* and *Schizosaccharomyces pombe* had already identified genes that upon deletion confer sensitivity to CP [39-41]. Genes surveyed by us included those operating in NER (*RAD10*), DNA damage tolerance (*RAD5, MMS2*, *REV3* and *RAD18*), interstrand crosslink repair (*PSO2*) and HR (*RAD51*). In general, the published relative CP sensitivities of the mutants investigated were confirmed (Figure S2, data not shown). Figure 2 summarizes the effect of NSC109268 on CP sensitivity of all tested deletion mutants, expressed as the factor by which the CP dose resulting in 50% or 10% survival can be reduced in the presence of NSC109268 (dose enhancement factor, DEF), see also Table S1 for data and gene product function. In Figure 3, selected dose response curves are shown.

In surveying a large number of isogenic mutants of haploid strain BY4741, we ruled out several pathways as primary targets of NSC109268 in causing cellular sensitization to CP. For example, *PSO2* is a gene known to be specifically involved in interstrand crosslink repair in yeast. CP is known to induce interstrand crosslinks eliciting cell killing in yeast [42] and as expected, we found the *pso2Δ* mutant strain to be hypersensitive to CP alone. The combination of NSC109268 and CP was highly synergistic in inducing cell death (Figure 2, Figure 3A) which ruled out the affected interstrand crosslink repair pathway as the target (and possibly also interstrand crosslinks as critical lesion). When testing *rad10Δ* mutant strain, defective in a key endonuclease component of NER, we found the strain to be hypersensitive to CP, as previously reported [39], and additional sensitization to CP by NSC109268 was still observed (Figure 2, Figure 3A). Similar results with *rev3Δ* and *rad30Δ* mutant strains argued against damage tolerance by translesion synthesis as the target of NSC109268 in mediating cellular sensitization to CP (Figure 2). Rtt101, a component of a novel ubiquitin ligase complex promoting replication through damaged DNA at stalled replication forks [43] could also be excluded (Figure 2). Similarly, by testing the CP-hypersensitive *rad50Δ* mutant, we concluded that the MRN/X (Mre11-Rad50-Xrs2) complex and its activity in DNA double-strand break repair is an unlikely target (Figure 2). 

As representative examples of the cell cycle checkpoint pathway, we selected mutants deleted for the DNA damage recognition protein Rad24 and the Rad53 kinase. A deletion of the latter is viable in an *SML1* deletion background. Both *sml1*∆ and *sml1*∆ *rad53∆* strains can be sensitized towards CP by NSC109268 to a similar extent (Figure 2, Figure 3B). Since *rad24∆* retained synergism as well, we did not obtain evidence for the checkpoint pathway being a critical target.

In revisiting CP uptake, we found combination treatment to remain synergistic in inducing cell killing in CP-resistant *ctr1Δ*, a CP uptake mutant (Figure 2). Similar results were obtained for *ccc2Δ*, deleted for the yeast ortholog of *ATP7B*, a P-type ATPase that mediates cellular CP efflux and counteracts CP lethality in mammals [5]. Together, these results confirm our previous notion that modification of CP transport is unlikely to be a mechanism by which NSC109268 mediates cellular sensitization to CP. 

As previously reported [39], mutant strain *rad5Δ*, defective in damage tolerance by TS, was found to be CP hypersensitive. Remarkably, we did not detect additional sensitization upon the administration of NSC109268 during CP treatment of *rad5Δ* cells (Figure 2, Figure 3C). This result was confirmed by detecting a similar absence of synergism in deletion mutants of other components of the Rad5 mediated TS pathway – *RAD18*, *UBC13* and *MMS2* (Figure 2, Figure 3C). As compared to wild-type, no or only a low degree of sensitization by NSC109268 beyond an additive effect was also found for deletions of the *RAD51, SGS1* and *PSY3*, encoding HR components that all are likely to play a role in damage tolerance by TS [28,44] (Figure 2, Figure 3D). 

We also tested a deletion of Rad59, an HR protein that stimulates single-strand annealing in complex with Rad52 [45]. Rad59 has been proposed to only participate in the canonical HR pathway but *not* in the TS pathway of damage tolerance [28]. The combination of CP and NSC109268 remained synergistic in inducing cell death in the *rad59Δ* strain (Figure 2, Figure 3D). This result implies that HR pathway *per se* may not be a crucial target of NSC109268 in mediating cellular sensitization to CP.

### NSC109268 enhances cisplatin-induced gene conversion and genome instability

In a diploid strain (D7, unrelated to BY4741), we tested the influence of CP and NSC109268 on survival and genomic instability events related to interchromosomal recombination. Strain D7 contains two detection systems for such events, the *ade2-119/ade2-40* and *trp5-12/trp5-27* heteroalleles [33]. Scoring red or pink sectored or pure colonies detects a wider range of events than the *trp5* gene conversion system, including chromosome loss events. 

**Figure 4:**
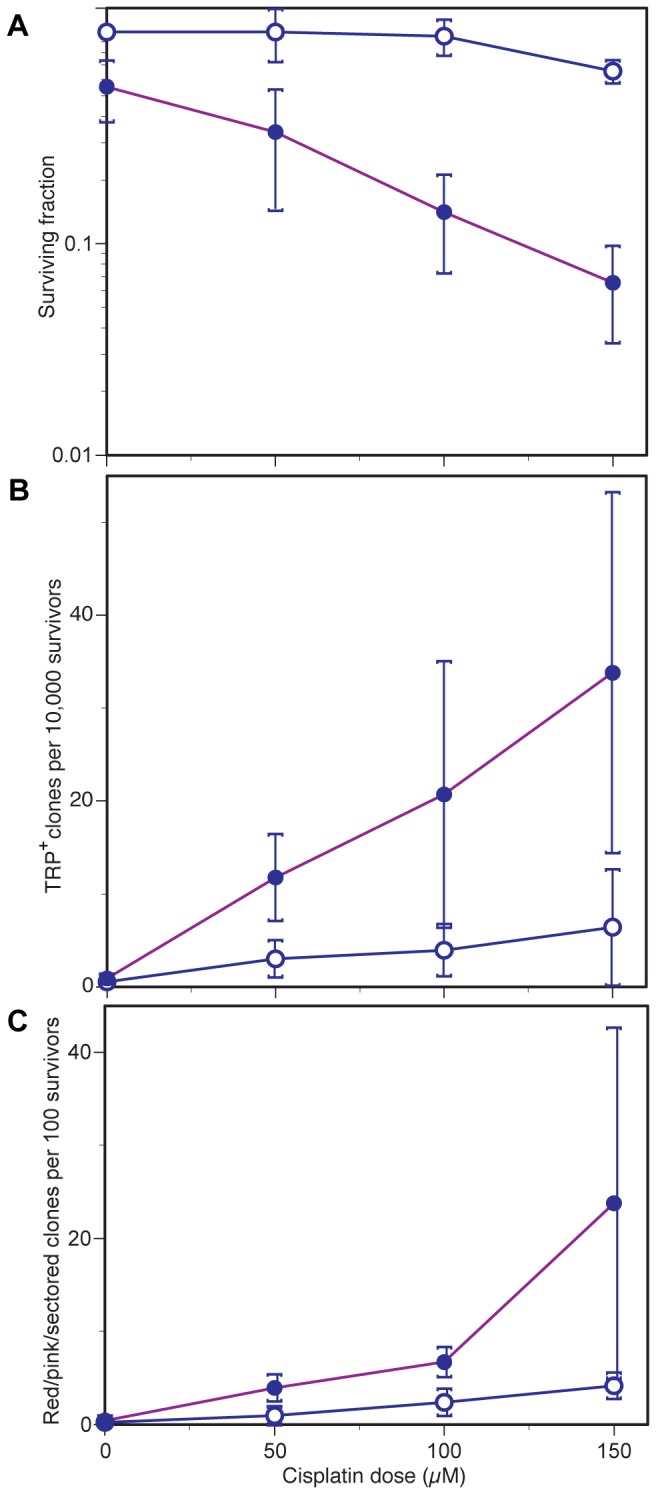
Effect of cisplatin and NSC109268 on survival and induced gene conversion/mitotic recombination in a diploid reporter strain. (A) Surviving fractions of colony forming cells, (B) frequency of TRP^+^ convertants, (C) frequency of red or pink sectored or pure clones of strain D7. Data were plotted as a function of CP dose, administered with or without added NSC109268 (8 µM). Values in B and C were corrected for spontaneous TRP^+^ and red/pink colony background frequencies.

For this diploid strain, synergistic interaction of CP and NSC109268 was verified for colony survival (Figure 4A). Frequency of CP-induced gene conversion in the *trp5* system and aberrant colony formation in the *ade2* system were increased in a dose-dependent manner (Figure 4B,C). Whereas NSC109268 had little effect on its own, it synergistically enhanced CP-induced recombination/aberrant colony frequency in either system. Since gene conversion and mitotic recombination events are dependent on HR [46], an inhibitory effect of NSC109268 on HR *per se* appears to be unlikely.

### No increased S phase extension by NSC109268 in cisplatin-treated *rad5Δ* mutant cells

**Figure 5:**
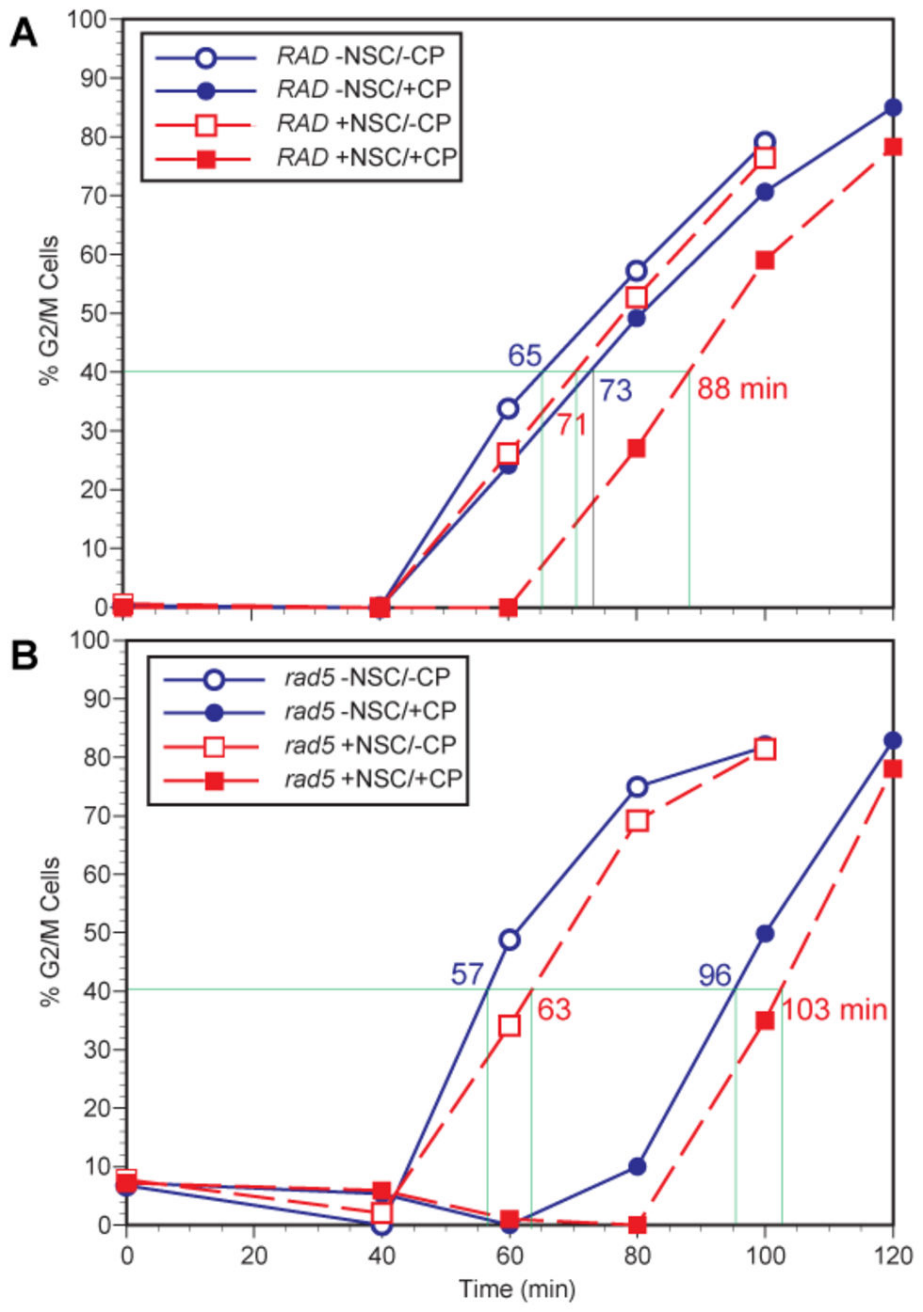
Flow cytometric analysis of DNA content during cell cycle progression of G1-synchronized cells. (A) Wild-type and (B) *rad5*∆ cells are compared following treatment with CP (80 μM), NSC109268 (0.7 µM) or the combination thereof. The indicated time in minutes to reach 40% G2/M phase cells is selected as a measure to compare S-phase extension in the different cultures. See Figure S3 for individual FACS profiles.

G1-synchronized haploid wild-type and isogenic *rad5Δ* strains were treated with CP alone or sequentially with CP and NSC109268 before release into nutrient medium to allow for cell cycle progression. By itself, the chosen low dose of NSC109268 caused only a brief delay of G1/S phase transition. To facilitate interpretation, we used a low CP dose that induced only a small effect on cell cycle kinetics in wild-type cells on its own. However, confirming previously published data [12], the combination of NSC109268 and CP resulted in a notable slow-down of S phase progression, as measured by a delayed increase in G2/M phase cells (Figure 5A, see Figure S3 for FACS profiles). Using the same CP dose, synchronized *rad5Δ* cells were delayed for G2/M entry as compared to untreated cells (Figure 5B). However, NSC109268 did not lead to any enhancement of this CP-dependent delay beyond the small effect attributable to NSC109268 alone, thus being additive at best (Figure 5B). Preliminary studies suggest that the same is true for the later occurring extensive G2/M arrest (data not shown). This is in marked contrast to the synergistic effect on cell cycle kinetics of NSC109268 in CP-treated wild-type cells (Figure 5A). Thus, cell cycle studies supported our survival analysis hinting at the Rad5 pathway of damage tolerance by TS as a target of NSC109268 in mediating cellular sensitization to CP. 

### Phosphatase inhibitory activity of NSC109268 is likely to mediate sensitization towards cisplatin

We wished to determine if the known activities of NSC109268 as 20S proteasome inhibitor [47] or as phosphatase 2Cα and 2A inhibitor [14] critically affect sensitization to CP by NSC109268. Bortezomib, a known proteasome inhibitor possibly mimicking NSC109268’s proteasome inhibitory activity, was not synergistic in inducing cell death (Figure S4A). Furthermore, NSC109268 and CP combination remained synergistic in *ump1Δ* (Figure 2, Table S1), deleted for a DNA-damage inducible chaperone involved in 20S proteasome maturation and required for UV resistance [48]. These results argue against *direct* proteasome inhibition by NSC109268 as critical for mediating sensitization to CP. 

Polyubiquitination of PCNA is an essential signal for activation of the Rad5 pathway. As an *indirect* consequence of proteasome inhibition by NSC109268 due to reduced ubiquitin recycling, we considered that free ubiquitin levels in NSC109268 + CP treated cells may be too low for PCNA polyubiquitination to occur efficiently [49]. This possibility, however, was unlikely since NSC109268 and CP combination remained synergistic in inducing cell death even if cells were overexpressing plasmid-encoded ubiquitin [50] (Figure S4B). Therefore, we found no evidence that proteasome inhibition by NSC109268 leads to cellular sensitization to CP, directly or indirectly through impairment of free ubiquitin levels. 

Next, we considered the phosphatase inhibitory activity of NSC109268. To test for the role of PP2C or PP2A inhibition by NSC109268 in CP sensitization, we first treated single mutant strains defective in Ptc2 or Ptc3, both classified as Ser/Thr protein phosphatase 2C family members, or Pph3, a type 2A-like protein phosphatase A. The combination of NSC109268 and CP was found to remain synergistic in inducing cell death in all single gene deletion mutants of haploid strain BY4741 (Figure 2). 

**Figure 6:**
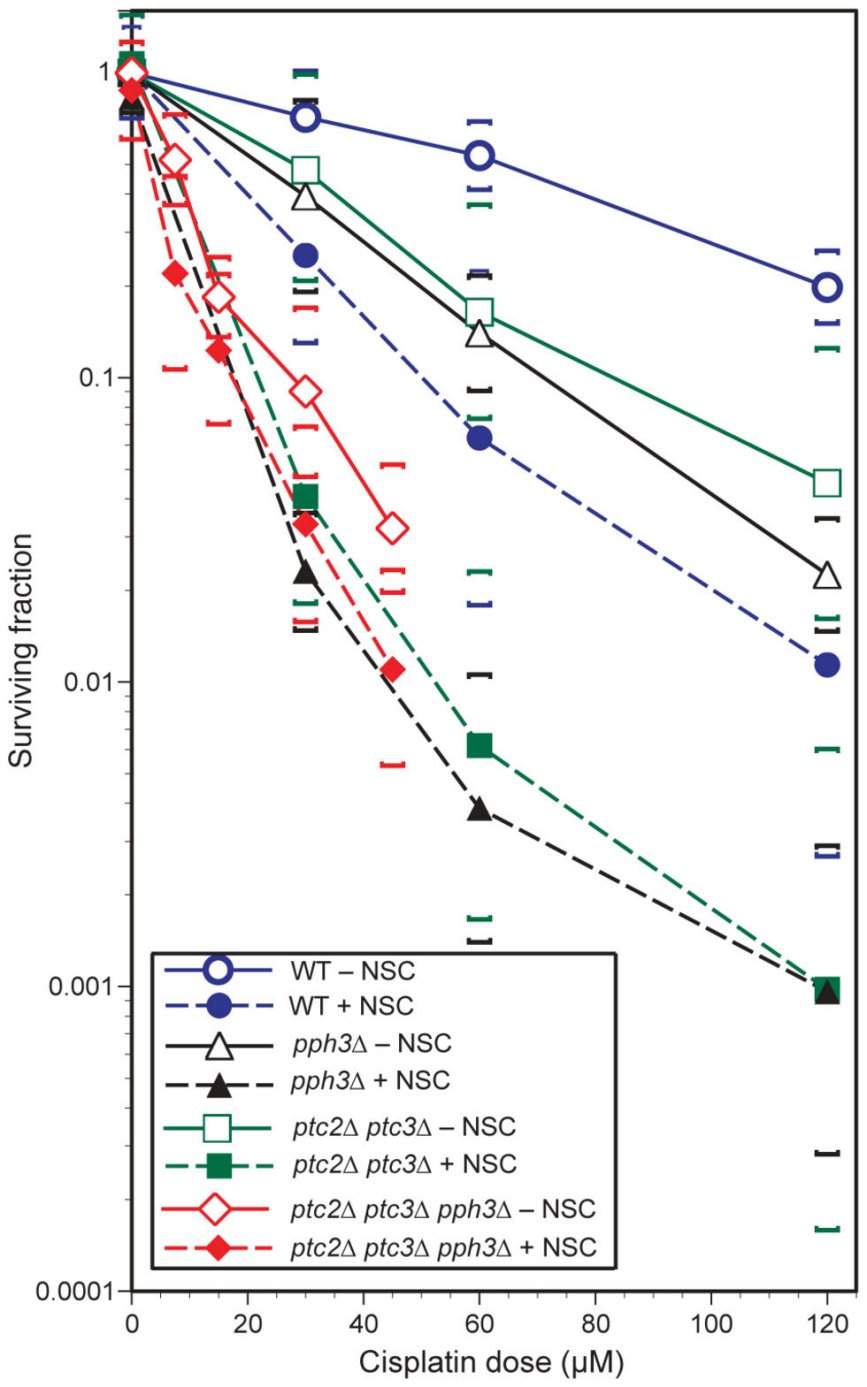
Influence of phosphatase deficiencies on NSC109268 in mediating cisplatin sensitization. Survival analyses of single, double and triple phosphatase gene deletion strains of yeast treated with CP, alone or in combination with NSC109268 (3 μM). Dose-response curves of YJK17 (WT), YJK26 (pph3∆), YJK24 (ptc2∆ptc3∆) and YJK70 (ptc2Δptc3Δpph3Δ) are shown. Surviving fractions are plotted as a function of CP dose, with or without NSC109268 (3 μM) present during treatment. Use of symbols is indicated in the figure.

Next, combinations of phosphatase mutations were studied in a different genetic background (YJK). As compared to BY4741, it should be noted that we were able to increase the dose enhancement effect in this strain background while reducing the lethality of NSC109268 alone. At 50%/10% CP survival, DEF between 3 and 5 were determined for WT, *pph3*∆ and *ptc2∆ptc3*∆ (Figure 2, Figure 6). However, whereas CP sensitivity without NSC109268 was notably enhanced in the triple mutant *ptc2Δptc3Δpph3Δ* synergism was greatly diminished, with DEF reduced to less than 2 (Figure 2, Figure 6). While confirming the known redundancy between the studied phosphatases [32] these results indicate the involvement of both phosphatase 2C or 2A inhibitory activities of NSC109268 in mediating CP sensitization. 

## Discussion

Among the platinum family of anti-cancer compounds, CP has been a very effective agent against a host of tumors such as those of the ovary and testis. However, its utility is severely restricted by dose limiting side effects and cellular resistance, either intrinsic or acquired during CP administration. We identified NSC109268 as a compound increasing cellular sensitivity to CP in *Saccharomyces cerevisiae* [12]. Enhancement of cellular sensitization to CP by NSC109268 was confirmed for CP-sensitive ovarian carcinoma 2008 cells and the derived CP-resistant line 2008/C13 [12]. Shown in both yeast and 2008/C13 cells, the increase in cellular sensitivity to CP by NSC109268 correlated with inhibition of S phase progression [12]. In *in vivo* studies with xenografted human head and neck carcinomas treated with CP alone, others had shown that a hindrance of S phase traversal correlated with CP responsiveness better than G2/M arrest [51].

In this study, we explored the possible targets of NSC109268 responsible for enhancing CP sensitivity using the systematic yeast gene deletion collection. We were guided by the concept that absence of the target should cause CP sensitivity but preclude additional sensitization by NSC109268. A similar strategy resulted in the identification of HR as the target of synergism between CP and camptothecin in yeast and human cancer cells [52]. Alterations in uptake or efflux of CP appeared unlikely to be the cause of sensitization since synergism was not diminished if CP was administered first, prior to treating with NSC109268 in CP-free buffer. This notion was further confirmed by mutant analysis. 

After investigating various DNA repair and tolerance pathways such as NER, DNA translesion synthesis or interstrand crosslink repair, we identified a DNA damage tolerance pathway, the Rad5 pathway, as critical determinant of the synergism between CP and NSC109268 in budding yeast. This pathway is believed to bypass DNA damage at replication forks, that have been stalled in S phase due to DNA adducts, by using the newly synthesized strand of the sister chromatid as a template (Figure S1) [28-31,53]. Fork regression or recombinative sister strand junction formation have been discussed as the underlying mechanisms in this process [29]. CP primarily causes bulky DNA adducts which lead to a slow down of the replication fork, eventually leaving behind single-stranded gaps to be filled by Rad5 mediated TS pathway, involving certain HR components [28,31]. Inhibition of Rad5 mediated lesion bypass activity by NSC109268 may also result in fork collapse leading to genetic instability – as shown here with reporter strain D7 – and finally cell death, thus mediating cellular sensitization to CP. It has been debated if the Rad5 mechanism is critical for S-phase progression in the presence of DNA damage, as suggested here, or acts predominantly in G2-phase to fill remaining single-stranded gaps [30,54-56]. The discrepancies in timing found in the literature may very well depend on level and kind of DNA damage. 

Our findings seem to fit particularly well with the model of filling of single-stranded gaps by template switch using a subset of recombination functions (Figure S1) [28,31]. We found proteins involved in initial PCNA monoubiquitination, such a Rad6 or Rad18, and in subsequent polyubiquitination, such as Rad5, Ubc13 or Mms2, to be required for CP+NSC109268 synergism. But we also identified HR proteins such as Rad51 or the junction-resolving enzyme Sgs1 as participants of the targeted mechanism(s). This includes Psy3, a member of the Shu complex proposed to participate in TS following strand invasion [44]. All of these proteins that are required for synergism of CP and NSC109268 are exclusively or non-exclusively involved in the TS pathway. 

Interestingly, the mutant group showing no or very reduced synergism did not include Rad59 which has been shown by others to play a role in canonical HR but not in the TS pathway [28]. We also demonstrated that CP-mediated and HR-dependent gene conversion events [46] are increased by NSC109268 and not decreased. (Current models of the Rad5 pathway do not involve the homologous chromosome in a template switch mechanism, so this result does not contradict the inhibition of the Rad5 pathway.) Based on these observations, we do not favor that NSC109268 acts as a general HR inhibitor to exert its function on CP sensitivity, however, a more rigorous demonstration is still required.

A screen for single gene deletion mutants of budding yeast conferring exquisite CP sensitivity had previously revealed *RAD5* and other key players of the same pathway as top hits [39]. In humans, this pathway was initially regarded as a tumor suppressor pathway since the essentially error-free bypass should counteract genetic instability [29,57]. On the other hand, the human ortholog of the yeast Rad5, HLTF, was reported to be overexpressed in radiation resistant recurrent human cervical carcinoma and a knockdown of HLTF in HeLa cells lead to a decrease in cellular proliferation [58]. Conceivably, targeting HLTF in chemotherapy-resistant cancers may lead to increase in cellular CP sensitivity. 

Upon investigating the known activities of NSC109268 as a 20S proteasome and phosphatase 2C and 2A inhibitor [13,14], we found no evidence for the importance of proteasome inhibition in mediating CP sensitivity in yeast. In contrast, our survival studies indicated 2C and 2A phosphatase inhibition by NSC109268 to be responsible for mediating CP sensitization. Analysis of the yeast single mutants and double mutants *ptc2Δ, ptc3Δ* (each one defective for functionally redundant PP2C phosphatases), *pph3Δ* (defective for PP2A-like phosphatase) compared to the triple phosphatase mutant *ptc2Δptc3Δpph3Δ* revealed a marked defect in the viability of *ptc2Δptc3Δpph3Δ* cells in the presence of CP, while neither of the single mutants were similarly sensitive to CP. These results suggested redundancy of phosphatase PP2C and phosphatase PP2A-like mediated pathways in CP damage responses, as was already reported for other agents such as hydroxyurea or methyl methanesulfonate [32]. If phosphatase redundancy is removed in the triple mutant, sensitization of CP-treated cells by NSC109268 is greatly reduced suggesting phosphatases as the relevant target.

Phosphatases Psy2 or Ptc2 and, in a redundant fashion, Ptc3 have been shown to be required for turning off the DNA damage-induced cell cycle checkpoint by dephosphorylating kinase Rad53 [59,60]. However, upon treatment of *rad53Δ* mutant (*sml1Δ* background), Rad53 was excluded as a major target of NSC109268 in mediating CP sensitization. Thus, a reduced ability to dephosphorylate Rad53 checkpoint kinase and to release checkpoint arrest is not responsible for the cellular sensitization to CP by NSC109268. 

Although the actual protein target(s) remain to be determined, this study underlines the critical importance of protein phosphatases in DNA damage responses that goes beyond recovery from checkpoint arrest. Even in a model organism such as yeast, significant gaps in our knowledge will need to be filled. For the first time, this study shows an influence of phosphatases on the Rad5 tolerance pathway. Additionally, the notion of intertwined HR and TS pathways is supported [28,30,31]. Interestingly, the triple phosphatase mutant *pph3Δptc2Δptc3Δ* used by us was reported to be defective in early steps of HR [32] that may also be necessary for sister chromatid junction formation and processing within the Rad5 TS pathway [28,30,31]. Interestingly, Rpa2, a subunit of the single-strand DNA binding complex Replication Protein A, represents another target candidate since its phosphorylation status, which may be enhanced by NSC109268-mediated phosphatase inhibition, is inversely correlated with RAD51 foci formation [61]. 

Taken together, our studies with NSC109268 in budding yeast demonstrate its potential as a cellular chemotherapy sensitizer, most likely by acting through the inhibition of dephosphorylation of critical protein(s) of the Rad5 pathway mediating CP damage tolerance during S phase. NSC109268 and related compounds may thus be especially valuable in CP combination therapy of the subset of cancers that developed CP resistance due to alterations within the Rad5 pathway. It remains to be seen if this is a mechanism of resistance that is widespread among cancer patients.

## Supporting Information

Figure S1 **Model for DNA damage tolerance by template switch.** Provoked by a bulky lesion (star) in the leading strand template, the proposed interplay of recombination proteins, PCNA ubiquitination and DNA polymerases in filling a single-stranded gap is depicted. Adapted from [28].
(PDF)

Figure S2 **Relative cisplatin sensitivity of haploid yeast mutants in agar diffusion assays.** For these semiquantitative streak tests, early-logarithmic phase BY4741 wild-type or mutant cell cultures were concentrated to 4x10^7^ cells/ml. Of these suspensions, 10 µl samples were streaked on YPD plates in a radial fashion, and 250 µl of 1.5 mM CP were pipetted in the circular center hole (0.5 inch diameter). Plates were kept at 4°C for 3 hours to allow for CP diffusion before incubating at 30°C. Streaks were photographed after 30 hours.
(PDF)

Figure S3 **Flow cytometric DNA profiles of G1-synchronized cells released into fresh medium after treatment with cisplatin, NSC109268 or both.** Wild-type (A) or *RAD5*-deleted cells (B) of BY4741 were synchronized with α-factor and then sequentially incubated with CP and NSC10268 before release into fresh YPD medium, as described in Material and Methods. Samples were taken for a period of 100 or 120 min past treatment. Proper control regimens (mock treatment, CP alone, NSC10268 alone) were applied for comparison and are indicated in the figure.
(PDF)

Figure S4 **Absence of evidence for direct of indirect influence of inhibition of protein degradation on sensitization to cisplatin by NSC109268.** (A) The absence of a non-additive effect of the combination of bortezomib and CP is shown by determining the survival of wild type cells (BY4741) after simultaneous treatment with CP and bortezomib (75, 338 or 375 μM) for 2 h. Surviving fractions of colony forming cells were plotted as a function of CP dose. (B) The absence of an influence of ubiquitin overexpression on the synergism of. CP and NSC109268 in inducing cell killing is shown. The dose responses of wild-type cells overexpressing ubiquitin after treatment with CP and with or without NSC109268 (3 μM) for 2 h are shown. Wild-type cells (SX46A *MATa RAD* *ade2* (*ochre*) *his3-532* *trp1-289* *ura3-52*) had been transformed with a plasmid carrying wild-type ubiquitin gene under the control of the copper inducible *CUP1* promoter (YEp96-*CUP1-UB*) [50], kindly provided by Dr. Mark Hochstrasser. Strains transformed with YEp96-CUP1-UB or vector plasmid were grown to early logarithmic phase in Trp-dropout medium. To overexpress ubiquitin, CuSO4 was added at 100 μM to the medium for 3 h before treatment and plating onto *–*Trp dropout plates.
(PDF)

Table S1 **Mutants studied for influence of NSC109268 on cisplatin sensitivity, ranked by dose enhancement factor (DEF) at 50% survival.** See also Figure 2.
(PDF)

## References

[1] Rabik CA, Dolan ME (2007) Molecular mechanisms of resistance and toxicity associated with platinating agents. Cancer Treat Rev 33: 9-23. doi:10.1016/j.ctrv.2006.09.006. PubMed: 17084534. 17084534 PMC1855222

[2] Kelland L (2007) The resurgence of platinum-based cancer chemotherapy. Nat Rev Cancer 7: 573-584. doi:10.1038/nrc2167. PubMed: 17625587. 17625587

[3] Galluzzi L, Senovilla L, Vitale I, Michels J, Martins I et al. (2012) Molecular mechanisms of cisplatin resistance. Oncogene 31: 1869-1883. doi:10.1038/onc.2011.384. PubMed: 21892204. 21892204

[4] Siddik ZH (2003) Cisplatin: mode of cytotoxic action and molecular basis of resistance. Oncogene 22: 7265-7279. doi:10.1038/sj.onc.1206933. PubMed: 14576837. 14576837

[5] Samimi G, Katano K, Holzer AK, Safaei R, Howell SB (2004) Modulation of the cellular pharmacology of cisplatin and its analogs by the copper exporters ATP7A and ATP7B. Mol Pharmacol 66: 25-32. doi:10.1124/mol.66.1.25. PubMed: 15213293. 15213293

[6] Andrews PA, Velury S, Mann SC, Howell SB (1988) cis-Diamminedichloroplatinum(II) accumulation in sensitive and resistant human ovarian carcinoma cells. Cancer Res 48: 68-73. PubMed: 3335000. 3335000

[7] Helleday T, Petermann E, Lundin C, Hodgson B, Sharma RA (2008) DNA repair pathways as targets for cancer therapy. Nat Rev Cancer 8: 193-204. doi:10.1038/nri2275. PubMed: 18256616. 18256616

[8] Wang D, Lippard SJ (2005) Cellular processing of platinum anticancer drugs. Nat Rev Drug Discov 4: 307-320. doi:10.1038/nrd1691. PubMed: 15789122. 15789122

[9] O'Connell D, Hopkins A, Roblin D (2007) Is it time to revisit the current R&D model? Int J Pharm Med 21: 339-345. doi:10.2165/00124363-200721050-00004.

[10] Chabner BA, Longo DL, editors (1996) Cancer Chemotherapy and Biotherapy: Principles and Practice. Philadelphia: Lippincott-Raven Publ.

[11] Zhang H, Siede W (2003) Validation of a novel assay for checkpoint responses: characterization of camptothecin derivatives in *Saccharomyces* *cerevisiae* . Mutat Res 527: 37-48. doi:10.1016/S0027-5107(03)00074-5. PubMed: 12787912. 12787912

[12] Jain D, Patel N, Shelton M, Basu A, Roque R et al. (2010) Enhancement of cisplatin sensitivity by NSC109268 in budding yeast and human cancer cells is associated with inhibition of S-phase progression. Cancer Chemother Pharmacol 66: 945-952. doi:10.1007/s00280-010-1246-8. PubMed: 20101404. 20101404

[13] Daniel KG, Gupta P, Harbach RH, Guida WC, Dou QP (2004) Organic copper complexes as a new class of proteasome inhibitors and apoptosis inducers in human cancer cells. Biochem Pharmacol 67: 1139-1151. doi:10.1016/j.bcp.2003.10.031. PubMed: 15006550. 15006550

[14] Rogers JP, Beuscher AE, Flajolet M, McAvoy T, Nairn AC et al. (2006) Discovery of protein phosphatase 2C inhibitors by virtual screening. J Med Chem 49: 1658-1667. doi:10.1021/jm051033y. PubMed: 16509582. 16509582 PMC2538531

[15] Shankar E, Basu S, Adkins B, Siede W, Basu A (2010) NSC109268 potentiates cisplatin-induced cell death in a p53-independent manner. J Mol Signal 5: 4. doi:10.1186/1750-2187-5-4. PubMed: 20459745. 20459745 PMC2889948

[16] Simon JA, Bedalov A (2004) Yeast as a model system for anticancer drug discovery. Nat Rev Cancer 4: 481-492. doi:10.1038/nrc1372. PubMed: 15170450. 15170450

[17] Sherman SE, Gibson D, Wang AH, Lippard SJ (1985) X-ray structure of the major adduct of the anticancer drug cisplatin with DNA: cis-[Pt(NH3)2(d(pGpG))]. Science 230: 412-417. doi:10.1126/science.4048939. PubMed: 4048939. 4048939

[18] Jung Y, Lippard SJ (2007) Direct cellular responses to platinum-induced DNA damage. Chem Rev 107: 1387-1407. doi:10.1021/cr068207j. PubMed: 17455916. 17455916

[19] Furuta T, Ueda T, Aune G, Sarasin A, Kraemer KH et al. (2002) Transcription-coupled nucleotide excision repair as a determinant of cisplatin sensitivity of human cells. Cancer Res 62: 4899-4902. PubMed: 12208738. 12208738

[20] Johnson SW, Laub PB, Beesley JS, Ozols RF, Hamilton TC (1997) Increased platinum-DNA damage tolerance is associated with cisplatin resistance and cross-resistance to various chemotherapeutic agents in unrelated human ovarian cancer cell lines. Cancer Res 57: 850-856. PubMed: 9041185. 9041185

[21] Mamenta EL, Poma EE, Kaufmann WK, Delmastro DA, Grady HL et al. (1994) Enhanced replicative bypass of platinum-DNA adducts in cisplatin-resistant human ovarian carcinoma cell lines. Cancer Res 54: 3500-3505. PubMed: 8012973. 8012973

[22] Hicks JK, Chute CL, Paulsen MT, Ragland RL, Howlett NG et al. (2010) Differential roles for DNA polymerases eta, zeta, and REV1 in lesion bypass of intrastrand versus interstrand DNA cross-links. Mol Cell Biol 30: 1217-1230. doi:10.1128/MCB.00993-09. PubMed: 20028736. 20028736 PMC2820889

[23] Friedberg EC, Walker GC, Siede W, Wood RD, Schultz RA et al. (2006) DNA Repair and Mutagenesis, 2nd Edition. Washington, D.C.: American Society of Microbiology Press.

[24] Chang DJ, Cimprich KA (2009) DNA damage tolerance: when it's OK to make mistakes. Nat Chem Biol 5: 82-90. doi:10.1038/nchembio.139. PubMed: 19148176. 19148176 PMC2663399

[25] Wu F, Lin X, Okuda T, Howell SB (2004) DNA polymerase zeta regulates cisplatin cytotoxicity, mutagenicity, and the rate of development of cisplatin resistance. Cancer Res 64: 8029-8035. doi:10.1158/0008-5472.CAN-03-3942. PubMed: 15520212. 15520212

[26] Doles J, Oliver TG, Cameron ER, Hsu G, Jacks T et al. (2010) Suppression of Rev3, the catalytic subunit of Polζ, sensitizes drug-resistant lung tumors to chemotherapy. Proc Natl Acad Sci U S A 107: 20786-20791. doi:10.1073/pnas.1011409107. PubMed: 21068376. 21068376 PMC2996428

[27] Albertella MR, Green CM, Lehmann AR, O'Connor MJ (2005) A role for polymerase eta in the cellular tolerance to cisplatin-induced damage. Cancer Res 65: 9799-9806. doi:10.1158/0008-5472.CAN-05-1095. PubMed: 16267001. 16267001

[28] Vanoli F, Fumasoni M, Szakal B, Maloisel L, Branzei D (2010) Replication and recombination factors contributing to recombination-dependent bypass of DNA lesions by template switch. PLOS Genet 6: e1001205 PubMed: 21085632. 21085632 10.1371/journal.pgen.1001205 PMC2978687

[29] Unk I, Hajdú I, Blastyák A, Haracska L (2010) Role of yeast Rad5 and its human orthologs, HLTF and SHPRH in DNA damage tolerance. DNA Repair (Amst) 9: 257-267. doi:10.1016/j.dnarep.2009.12.013. PubMed: 20096653. 20096653

[30] Chatterjee B, Siede W (2013) Replicating damaged DNA in eukaryotes. Cold Spring Harb Perspect Biol. In press 10.1101/cshperspect.a019836 PMC3839612 24296172

[31] Branzei D (2011) Ubiquitin family modifications and template switching. FEBS Lett 585: 2810-2817. doi:10.1016/j.febslet.2011.04.053. PubMed: 21539841. 21539841

[32] Kim JA, Hicks WM, Li J, Tay SY, Haber JE (2011) Protein phosphatases Pph3, Ptc2, and Ptc3 play redundant roles in DNA double-strand break repair by homologous recombination. Mol Cell Biol 31: 507-516. doi:10.1128/MCB.01168-10. PubMed: 21135129. 21135129 PMC3028631

[33] Zimmermann FK, Kern R, Rasenberger H (1975) A yeast strain for simultaneous detection of induced mitotic crossing-over, mitotic gene conversion and reverse mutation. Mutat Res 28: 381-388. doi:10.1016/0027-5107(75)90232-8.

[34] Amberg DC, Burke DJ, Strathern JN (2005) Methods in Yeast Genetics: A Cold Spring Harbor Laboratory Course Manual, 2005 Edition. Cold Spring Harbor, NY: Cold Spring Harbor Laboratory Press.

[35] Fortuna M, Jaoa Sousa M, Côrte-Real M, Leão C (2001) Cell cycle analysis of yeasts. Current Protoc Cytometry Unit 11: 13 PubMed: 18770687. 10.1002/0471142956.cy1113s13 18770687

[36] Ishida S, Lee J, Thiele DJ, Herskowitz I (2002) Uptake of the anticancer drug cisplatin mediated by the copper transporter Ctr1 in yeast and mammals. Proc Natl Acad Sci U_S_A 99: 14298-14302. doi:10.1073/pnas.162491399. PubMed: 12370430. 12370430 PMC137878

[37] Holzer AK, Manorek GH, Howell SB (2006) Contribution of the major copper influx transporter CTR1 to the cellular accumulation of cisplatin, carboplatin, and oxaliplatin. Mol Pharmacol 70: 1390-1394. doi:10.1124/mol.106.022624. PubMed: 16847145. 16847145

[38] Holzer AK, Howell SB (2006) The internalization and degradation of human copper transporter 1 following cisplatin exposure. Cancer Res 66: 10944-10952. doi:10.1158/0008-5472.CAN-06-1710. PubMed: 17108132. 17108132

[39] Wu HI, Brown JA, Dorie MJ, Lazzeroni L, Brown JM (2004) Genome-wide identification of genes conferring resistance to the anticancer agents cisplatin, oxaliplatin, and mitomycin C. Cancer Res 64: 3940-3948. doi:10.1158/0008-5472.CAN-03-3113. PubMed: 15173006. 15173006

[40] Simon JA, Szankasi P, Nguyen DK, Ludlow C, Dunstan HM et al. (2000) Differential toxicities of anticancer agents among DNA repair and checkpoint mutants of *Saccharomyces* *cerevisiae* . Cancer Res 60: 328-333. PubMed: 10667584. 10667584

[41] Perego P, Zunino F, Carenini N, Giuliani F, Spinelli S et al. (1998) Sensitivity to cisplatin and platinum-containing compounds of *Schizosaccharomyces* *pombe* rad mutants. Mol Pharmacol 54: 213-219. PubMed: 9658208. 9658208 10.1124/mol.54.1.213

[42] Grossmann KF, Ward AM, Matkovic ME, Folias AE, Moses RE (2001) *S.* *cerevisiae* has three pathways for DNA interstrand crosslink repair. Mutat Res 487: 73-83. doi:10.1016/S0921-8777(01)00106-9. PubMed: 11738934. 11738934

[43] Zaidi IW, Rabut G, Poveda A, Scheel H, Malmström J et al. (2008) Rtt101 and Mms1 in budding yeast form a CUL4^DDB1^-like ubiquitin ligase that promotes replication through damaged DNA. EMBO Rep 9: 1034-1040. doi:10.1038/embor.2008.155. PubMed: 18704118. 18704118 PMC2572122

[44] Ball LG, Zhang K, Cobb JA, Boone C, Xiao W (2009) The yeast Shu complex couples error-free post-replication repair to homologous recombination. Mol Microbiol 73: 89-102. doi:10.1111/j.1365-2958.2009.06748.x. PubMed: 19496932. 19496932

[45] Davis AP, Symington LS (2001) The yeast recombinational repair protein Rad59 interacts with Rad52 and stimulates single-strand annealing. Genetics 159: 515-525. PubMed: 11606529. 11606529 10.1093/genetics/159.2.515 PMC1461847

[46] Haynes RH, Kunz BA (1981) DNA repair and mutagenesis in yeast. In: Strathern JN Jones EW Broach JR The Molecular Biology of the Yeast Saccharomyces. Cold Spring Harbor, N.Y.: Cold Spring Harbor Laboratory Publishing pp. 371-414.

[47] Daniel KG, Chen D, Yan B, Dou QP (2007) Copper-binding compounds as proteasome inhibitors and apoptosis inducers in human cancer. Front Biosci 12: 135-144. doi:10.2741/2054. PubMed: 17127289. 17127289

[48] Mieczkowski P, Dajewski W, Podlaska A, Skoneczna A, Ciesla Z et al. (2000) Expression of *UMP1* is inducible by DNA damage and required for resistance of *S.* *cerevisiae* cells to UV light. Curr Genet 38: 53-59. doi:10.1007/s002940000136. PubMed: 10975253. 10975253

[49] Gong J, Siede W (2011) Influence of deubiquitinating enzymes on mutagenesis in *Saccharomyces* *cerevisiae*. Internet. J Microbiol 9: 2.

[50] Swaminathan S, Amerik AY, Hochstrasser M (1999) The Doa4 deubiquitinating enzyme is required for ubiquitin homeostasis in yeast. Mol Cell Biol 10: 2583-2594. doi:10.1091/mbc.10.8.2583. PubMed: 10436014. PMC25490 10436014

[51] Jäckel M, Köpf-Maier P (1991) Influence of cisplatin on cell-cycle progression in xenografted human head and neck carcinomas. Cancer Chemother Pharmacol 27: 464-471. doi:10.1007/BF00685161. PubMed: 2013116. 2013116

[52] van Waardenburg RC, de Jong LA, van Delft F, van Eijndhoven MA, Bohlander M et al. (2004) Homologous recombination is a highly conserved determinant of the synergistic cytotoxicity between cisplatin and DNA topoisomerase I poisons. Mol Cancer Ther 3: 393-402. doi:10.4161/cbt.3.4.733. PubMed: 15078982. 15078982

[53] Minca EC, Kowalski D (2011) Replication fork stalling by bulky DNA damage: localization at active origins and checkpoint modulation. Nucleic Acids Res 39: 2610-2623. doi:10.1093/nar/gkq1215. PubMed: 21138968. 21138968 PMC3074140

[54] Karras GI, Jentsch S (2010) The *RAD6* DNA damage tolerance pathway operates uncoupled from the replication fork and is functional beyond S phase. Cell 141: 255-267. doi:10.1016/j.cell.2010.02.028. PubMed: 20403322. 20403322

[55] Daigaku Y, Davies AA, Ulrich HD (2010) Ubiquitin-dependent DNA damage bypass is separable from genome replication. Nature 465: 951-955. doi:10.1038/nature09097. PubMed: 20453836. 20453836 PMC2888004

[56] Minca EC, Kowalski D (2010) Multiple Rad5 activities mediate sister chromatid recombination to bypass DNA damage at stalled replication forks. Mol Cell 38: 649-661. doi:10.1016/j.molcel.2010.03.020. PubMed: 20541998. 20541998 PMC2887677

[57] Lin JR, Zeman MK, Chen JY, Yee MC, Cimprich KA (2011) SHPRH and HLTF act in a damage-specific manner to coordinate different forms of postreplication repair and prevent mutagenesis. Mol Cell 42: 237-249. doi:10.1016/j.molcel.2011.02.026. PubMed: 21396873. 21396873 PMC3080461

[58] Cho S, Cinghu S, Yu JR, Park WY (2011) Helicase-like transcription factor confers radiation resistance in cervical cancer through enhancing the DNA damage repair capacity. J Cancer Res Clin Oncol 137: 629-637. doi:10.1007/s00432-010-0925-5. PubMed: 20535496. 20535496 PMC11828077

[59] Leroy C, Lee SE, Vaze MB, Ochsenbein F, Guerois R et al. (2003) PP2C phosphatases Ptc2 and Ptc3 are required for DNA checkpoint inactivation after a double-strand break. Mol Cell 11: 827-835. doi:10.1016/S1097-2765(03)00058-3. PubMed: 12667463. 12667463

[60] O'Neill BM, Szyjka SJ, Lis ET, Bailey AO, Yates JR 3rd et al. (2007) Pph3-Psy2 is a phosphatase complex required for Rad53 dephosphorylation and replication fork restart during recovery from DNA damage. Proc Natl Acad Sci USA 104: 9290-9295. 17517611 10.1073/pnas.0703252104 PMC1890487

[61] Liaw H, Lee D, Myung K (2011) DNA-PK-dependent RPA2 hyperphosphorylation facilitates DNA repair and suppresses sister chromatid exchange. PLOS ONE 6: e21424. doi:10.1371/journal.pone.0021424. PubMed: 21731742. 21731742 PMC3120867

