# Supplementary figures and images for: Rad5 Template Switch Pathway of DNA Damage Tolerance Determines Synergism between Cisplatin and NSC109268 in Saccharomyces cerevisiae
Source: PLoS One. 2013 Oct 10;8(10):e77666. doi: 10.1371/journal.pone.0077666 (PMC3795065; doi:10.1371/journal.pone.0077666)

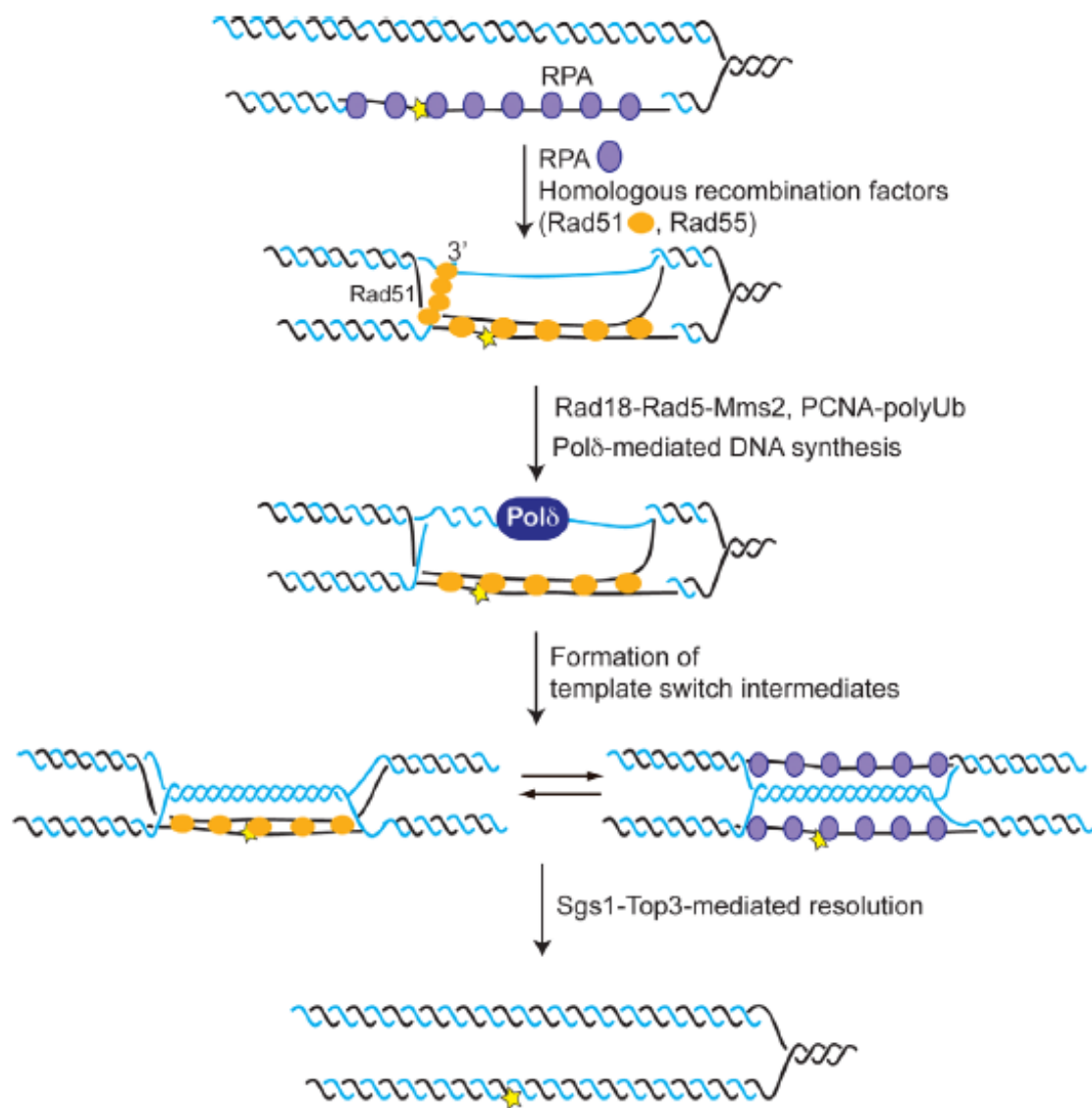

Supplement: Figure S1 — Model for DNA damage tolerance by template switch. Provoked by a bulky lesion (star) in the leading strand template, the proposed interplay of recombination proteins, PCNA ubiquitination and DNA polymerases in filling a single-stranded gap is depicted. Adapted from [28]. (PDF) [file pone.0077666.s001.pdf]

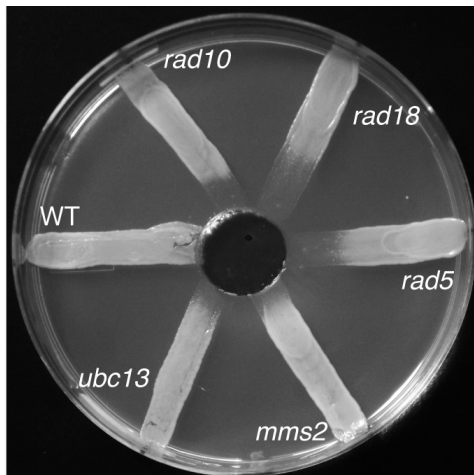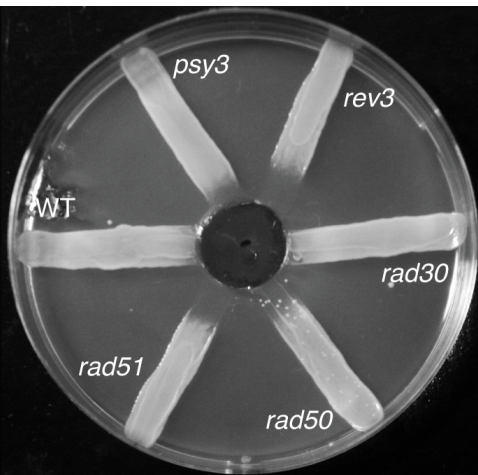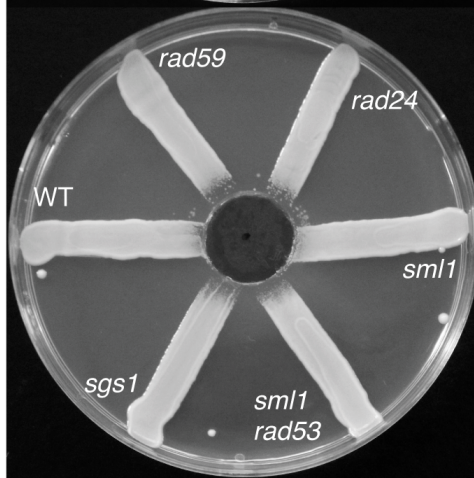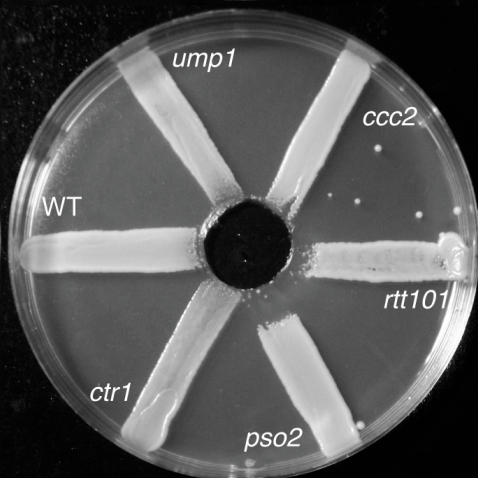

Supplement: Figure S2 — Relative cisplatin sensitivity of haploid yeast mutants in agar diffusion assays. For these semiquantitative streak tests, early-logarithmic phase BY4741 wild-type or mutant cell cultures were concentrated to 4x10^7^ cells/ml. Of these suspensions, 10 µl samples were streaked on YPD plates in a radial fashion, and 250 µl of 1.5 mM CP were pipetted in the circular center hole (0.5 inch diameter). Plates were kept at 4°C for 3 hours to allow for CP diffusion before incubating at 30°C. Streaks were photographed after 30 hours. (PDF) [file pone.0077666.s002.pdf]

**WT**

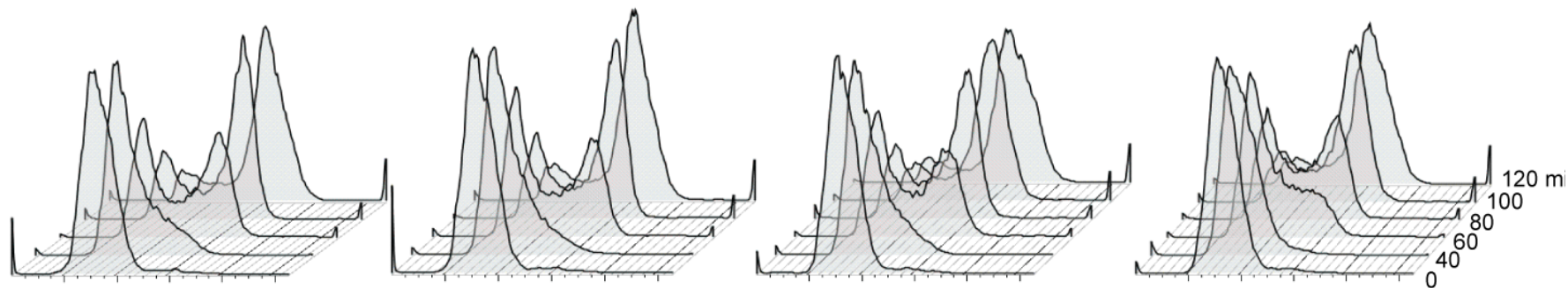

***rad5* $\Delta$**

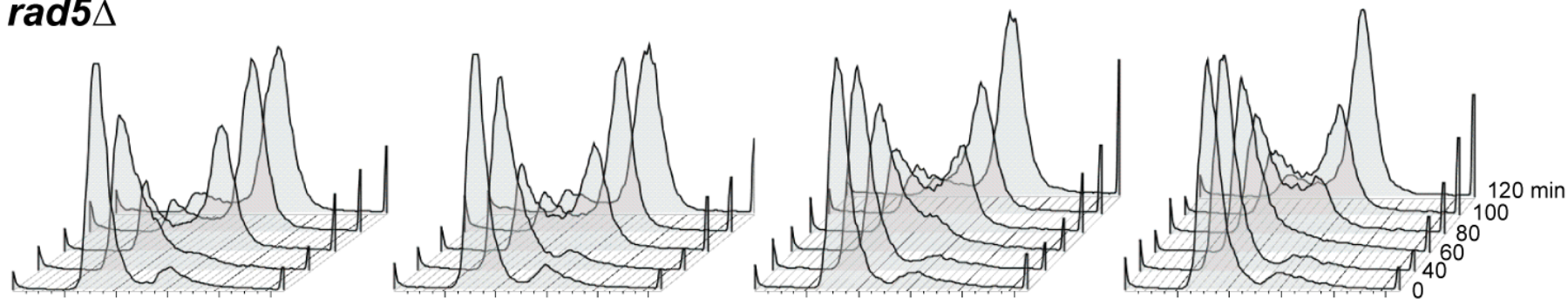

– CP  
– NSC109268

– CP  
+ NSC109268

+ CP  
– NSC109268

+ CP  
+ NSC109268

Supplement: Figure S3 — Flow cytometric DNA profiles of G1-synchronized cells released into fresh medium after treatment with cisplatin, NSC109268 or both. Wild-type (A) or RAD5-deleted cells (B) of BY4741 were synchronized with α-factor and then sequentially incubated with CP and NSC10268 before release into fresh YPD medium, as described in Material and Methods. Samples were taken for a period of 100 or 120 min past treatment. Proper control regimens (mock treatment, CP alone, NSC10268 alone) were applied for comparison and are indicated in the figure. (PDF) [file pone.0077666.s003.pdf]

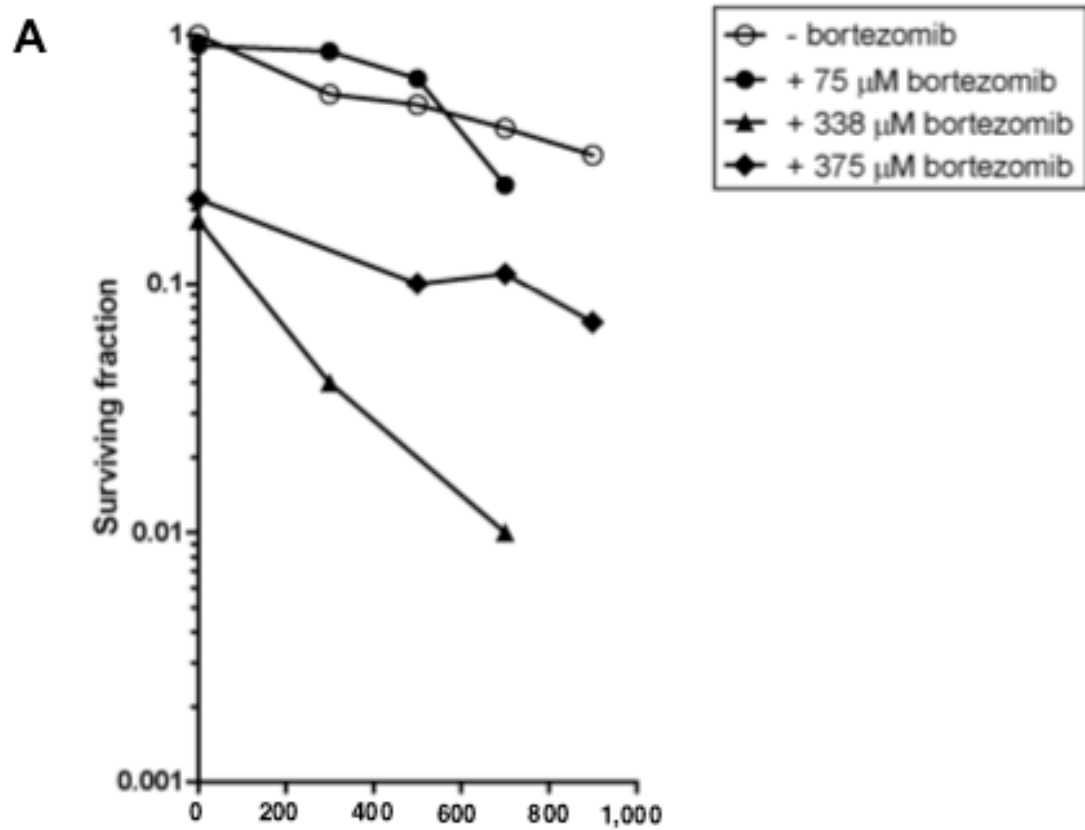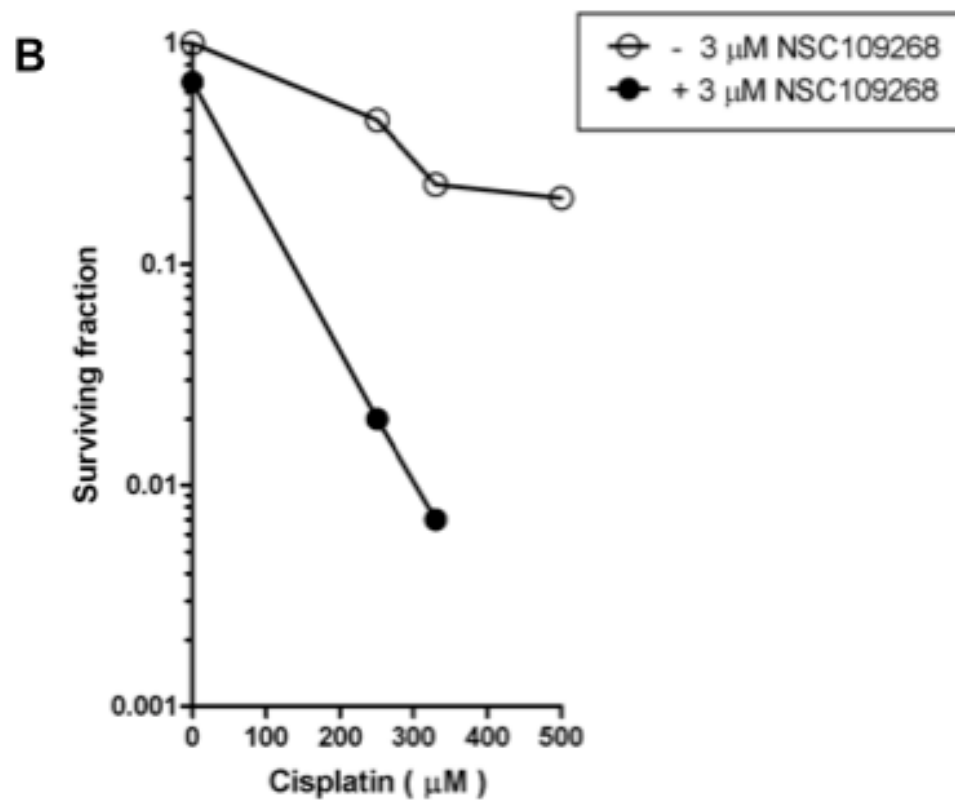

Supplement: Figure S4 — Absence of evidence for direct of indirect influence of inhibition of protein degradation on sensitization to cisplatin by NSC109268. (A) The absence of a non-additive effect of the combination of bortezomib and CP is shown by determining the survival of wild type cells (BY4741) after simultaneous treatment with CP and bortezomib (75, 338 or 375 μM) for 2 h. Surviving fractions of colony forming cells were plotted as a function of CP dose. (B) The absence of an influence of ubiquitin overexpression on the synergism of. CP and NSC109268 in inducing cell killing is shown. The dose responses of wild-type cells overexpressing ubiquitin after treatment with CP and with or without NSC109268 (3 μM) for 2 h are shown. Wild-type cells (SX46A MATa RAD ade2 (ochre) his3-532 trp1-289 ura3-52) had been transformed with a plasmid carrying wild-type ubiquitin gene under the control of the copper inducible CUP1 promoter (YEp96-CUP1-UB) [50], kindly provided by Dr. Mark Hochstrasser. Strains transformed with YEp96-CUP1-UB or vector plasmid were grown to early logarithmic phase in Trp-dropout medium. To overexpress ubiquitin, CuSO4 was added at 100 μM to the medium for 3 h before treatment and plating onto –Trp dropout plates. (PDF) [file pone.0077666.s004.pdf]
