# Supplementary material for: Rad5 Template Switch Pathway of DNA Damage Tolerance Determines Synergism between Cisplatin and NSC109268 in Saccharomyces cerevisiae
Source: PLoS One. 2013 Oct 10;8(10):e77666. doi: 10.1371/journal.pone.0077666 (PMC3795065; doi:10.1371/journal.pone.0077666)
Supplement: Table S1 — Mutants studied for influence of NSC109268 on cisplatin sensitivity, ranked by dose enhancement factor (DEF) at 50% survival. See also Figure 2. (PDF) [file pone.0077666.s005.pdf]

**Gene deletion**	**Function/pathway of inactivated gene product**	**DEF (50% survival)**	**DEF (10% survival)**
**BY4741 strain background**			
pso2	Interstrand cross-link repair	5.9	6.0
pph3	Protein phosphatase	3.2	2.5
ctr1	Copper transporter	2.9	3.0
rad30	Translesion synthesis	2.8	2.6
ump1	Chaperone required for 20S proteasome maturation	2.8	2.4
ptc3	Protein phosphatase	2.7	3.0
ccc2	Copper transporter	2.6	2.0
sml1	Ribonucleotide reductase inhibitor	2.5	2.0
sml1 rad53	Checkpoint kinase (*sml1* deletion required for viability)	2.5	1.5
WT	Wild type	2.5	1.80
rad59	Homologous recombination, single-strand annealing	2.1	1.9
rad50	MRN/X- repair complex	1.9	1.7
rad24	Checkpoint protein, DNA damage detection	1.9	1.4
ptc2	Protein phosphatase	1.8	1.9
rev3	Translesion synthesis	1.8	1.9
rad10	Nucleotide excision repair	1.7	1.5
rtt101	Ubiquitin-ligase component, promoting replication through damaged DNA	1.6	3.3
psy3	Shu complex component, promoting homologous recombination	1.2	1.1
sgs1	RecQ-like DNA helicase	1.2	1.1
rad18	DNA damage tolerance by template switch(TS) and translesion synthesis	1.2	1.0
rad51	Homologous recombination	1.1	1.2
ubc13	DNA damage tolerance (TS)	1.1	1.0
mms2	DNA damage tolerance (TS)	1.1	1.0
rad5	DNA damage tolerance (TS)	0.9	1.0
**YJK17 strain background**			
pph3	Protein phosphatase	5.2	4.0
WT	Wild type	4.2	3.0

ptc2 ptc3	Protein phosphatases	4.1	3.8
ptc2 ptc3 pph3	Protein phosphatases	1.9	1.6
